# Unusual Cause of Intestinal Obstruction: Left Paraduodenal Hernia

**DOI:** 10.1155/2012/529246

**Published:** 2012-03-04

**Authors:** Sami Akbulut

**Affiliations:** Department of Surgery, Diyarbakir Education and Research Hospital, 21400 Diyarbakir, Turkey

## Abstract

Internal intestinal hernia has been defined as bulging of the intestines through a normal or an abnormal peritoneal or mesenteric opening. Paraduodenal hernias comprise 30%–53% of all internal intestinal herniations and account for 0.2%–0.9% of all bowel obstructions. In this paper, we aimed to present a male patient of 42 years of age who developed intestinal obstruction due to left paraduodenal hernias. Left paraduodenal hernia should be taken into consideration in the differential diagnosis in patients of relatively younger ages with no prior abdominal surgery who frequently have bowel obstruction episodes.

## 1. Introduction

Internal intestinal hernia has been defined as bulging of the intestines through a normal or an abnormal peritoneal or mesenteric opening. This clinical entity can occur for congenital or acquired reasons and accounts for around 1%–5.8% of all intestinal obstructions [1–3]. Although the intestinal herniations are divided into distinct subgroups based on the localizations, it is the paraduedonal hernias which are responsible in nearly half of the patients. Also known as Treitz's hernia, the paraduedonal herniations eventuate from abnormal rotation of intestine. Paraduedonal hernias comprise 30%–53% of all intestinal herniations and account for 0.2%–0.9% of all bowel obstructions [1–4]. In this study, we aimed to present a male patient of 42 years of age who developed intestinal obstruction due to left paraduedonal hernias.

## 2. Case Presentation

**Figure 1:**
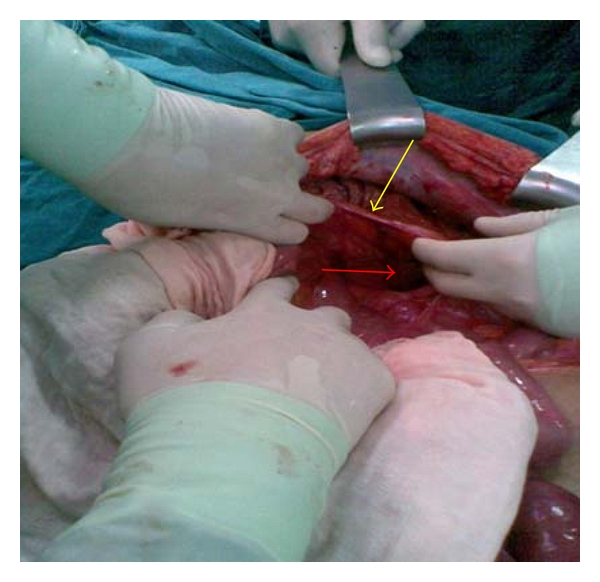
Left paraduedonal hernia sac. The red arrow points to the hernia sac, while the yellow arrow indicates inferior mesenteric vein, constituting a wall of the hernial sac.

A 42-year-old male patient was admitted to our emergency department with the complaints of nausea, vomiting, and abdominal pain that had lasted for 3 days and had shown progressive worsening. The patient especially expressed that the pain had intensified around the umbilical region and that he had been unable to pass stool or gas. The patient's history revealed no systemic disease or prior operation. As for the vital signs of the patient, the blood pressure, the pulse rate, and the body temperature were measured to be 130/85 mmHg, 83 beats/min and 37.3°C, respectively. Marked abdominal distension and rebound tenderness were detected in the physical examination performed, whereas no abdominal guarding was observed. In standing abdominal direct X-ray, widespread air-fluid levels were observed. While no pathological finding was detected other than mild uremia in the blood chemistry, leukocyte counts was reported to be 16.450/mm^3^. We were not able to obtain ultrasonographic or contrast-enhanced abdominal CT evaluation because of the fact that the patient was admitted to our emergency department at 11.00 pm. Given the physical examination findings, plain X-ray imaging and the absence of any prior surgery in the case history, the patient was taken into surgery considering that widespread peritonitis was likely to exist due to a possible congenital brid or a perforated appendicitis with delayed diagnosis. We observed, in the surgical exploration via midline incision, that all of the small intestinal segments except a distal ileal portion of 50–60 cm length was stuck in the sac of a left paraduedonal hernia. The inferior mesenteric vein constituted the anterior portion of the hernial sac from the orifice (Figure 1). After reducing the intestinal segments in the hernial sac into the abdominal cavity, IMV was released alongside its length. Then followed sealing off the orifice of the sac with sutures. The patient was discharged from the hospital on the 5th day after surgery without any complication.

## 3. Discussion

Intestinal hernias are entitled according to the orifices they pass through or to the position of the herniating organ. Based on the former definition, the most commonly seen hernia types are as follows: paraduedonal, transmesenteric, pericecal, intersigmoid, supravesical, foramen Winslow, retroanastomotic, and omental hernias. Paraduedonal hernia was first defined by Treitz in 1857. Paraduedonal hernia is the most common form among intestinal hernias, accounting for 30%–53% of all cases. Paraduedonal hernias result from abnormal rotation of the midgut during embryonic development and can be divided into two subtypes, left and right paraduedonal hernias, according to their distinct pathogenesis and the resultant anatomical derangement. 75% of them are located on the left side in the Landzert's paraduedonal fossa [3, 5]. Around 50% of the patients with paraduedonal hernias had episodes of intestinal obstruction in certain periods of their lives. The symptoms seen in these cases can range from temporary colicky pain in abdomen to the signs of intestinal obstruction. The remaining 50% of the cases follow an asymptomatic clinical course and are diagnosed incidentally [1]. Imaging methods play a pivotal role in the diagnosis of intestinal herniations. Plain X-rays can yield information regarding the intestinal segment from which the herniation stems and extension of the intestinal obstruction, while abdominal CT shows dislocated, distended, expanded, and gathered small intestinal segments [1, 5]. Moreover, CT can also indicate the dislocations of mesenteric vascular structures. Recently, diagnostic laparoscopy has provided the abilities of both verification of the diagnosis and simultaneous surgical intervention in cases that could not be diagnosed by radiological methods. The basic principles applied in the treatments of any kinds of herniations also hold true in the treatment of paraduedonal herniations. These are the repair of the defect and the resection of the hernial sac at times when reduction of incarcerated intestinal segments is necessary.

In conclusion, left paraduedonal hernia is one of the rare causes of small bowel obstruction. It should be taken into consideration in the differential diagnosis in patients of relatively younger ages with no prior abdominal surgery who frequently have bowel obstruction episodes.
